# ATF3 promotes ferroptosis in sorafenib-induced cardiotoxicity by suppressing Slc7a11 expression

**DOI:** 10.3389/fphar.2022.904314

**Published:** 2022-09-23

**Authors:** Yilan Li, Jingru Yan, Qianqian Zhao, Yan Zhang, Yao Zhang

**Affiliations:** ^1^ Department of Cardiology, The Second Affiliated Hospital of Harbin Medical University, Harbin, China; ^2^ Key Laboratory of Myocardial Ischemia, Ministry of Education, Harbin Medical University, Harbin, China; ^3^ Department of Oncology, The Second Affiliated Hospital of Harbin Medical University, Harbin, China

**Keywords:** sorafenib, cardiotoxicity, ferroptosis, SLC7A11, system xc-, ATF3

## Abstract

Sorafenib is the unique recommended molecular-targeted drug for advanced hepatocellular carcinoma, but its clinical use is limited due to cardiotoxicity. As sorafenib is an efficient ferroptosis inducer, the pathogenesis of this compound to ferroptosis-mediated cardiotoxicity is worth further study. Mice were administered 30 mg/kg sorafenib intraperitoneally for 2 weeks to induce cardiac dysfunction and Ferrostatin-1 (Fer-1) was used to reduce ferroptosis of mice with sorafenib-induced cardiotoxicity. Sorafenib reduced levels of anti-ferroptotic markers involving Slc7a11 and glutathione peroxidase 4 (GPX4), increased malonaldehyde malondialdehyde, apart from causing obvious mitochondria damage, which was alleviated by Fer-1. *In vitro* experiments showed that Fer-1 inhibited lipid peroxidation and injury of H9c2 cardiomyoblasts induced by sorafenib. Both *in vitro* and *in vivo* experiments confirmed that the expression of Slc7a11 was down regulated in sorafenib-induced cardiotoxicity, which can be partially prevented by treatment with Fer-1. Overexpression of Slc7a11 protected cells from ferroptosis, while knock-down of Slc7a11 made cardiomyoblasts sensitive to ferroptosis caused by sorafenib. Finally, by comparing data from the GEO database, we found that the expression of ATF3 was significantly increased in sorafenib treated human cardiomyocytes. In addition, we demonstrated that ATF3 suppressed Slc7a11 expression and promoted ferroptosis. Based on these findings, we concluded that ATF3/Slc7a11 mediated ferroptosis is one of the key mechanisms leading to sorafenib-induced cardiotoxicity. Targeting ferroptosis may be a novel therapeutic approach for preventing sorafenib-induced cardiotoxicity in the future.

## 1 Introduction

Sorafenib is a multi-targeted tyrosine kinase inhibitors (TKIs) of growth factor receptors—the most important of which are the platelet-derived growth factor receptor (PDGFR), vascular endothelial growth factor receptors (VEGFR), v-raf murine sarcoma viral oncogene homolog B1 (BRAF) and v-raf-1 murine leukaemia viral oncogene homolog 1 (RAF1) (Chen et al., 2008). Sorafenib is used in the treatment of advanced hepatocellular carcinoma and metastatic renal cell carcinoma (Pal et al., 2020; Ricke et al., 2021). However, the clinical application of sorafenib is limited by its cardiotoxic effects, including degenerative left ventricular dysfunction and congestive heart failure (Zamorano et al., 2016).

Notably, the molecular mechanism underlining sorafenib-induced cardiotoxicity remains unclear. Several mechanisms have been reported, including disruption of VEGF-VEGFR signalling through the inhibition of circulating VEGF and PDGFRs and inhibition of the rapidly accelerated fibrosarcoma (RAF)/extracellular signal-regulated kinase (ERK) pathway (Chen et al., 2008). Recent studies have shown that sorafenib can induce oxidative stress in H9c2 cardiomyoblasts through mitochondrial injury and oxygen species (ROS) production, etc. (Will et al., 2008; Hasinoff and Patel, 2010; Ma et al., 2020). Further research is needed to provide more options for preventing sorafenib-induced cardiotoxicity.

Recently, sorafenib was identified as a ferroptosis inducer in hepatocellular carcinoma, a newly recognized form of iron-dependent cell death (Louandre et al., 2013; Sun et al., 2021). Ferroptosis is characterized by the accumulation of iron-dependent lipid peroxidation products and increased ROS and oxidative stress is considered to be a principal cause of ferroptosis (Dixon et al., 2012).

Ferroptosis was induced by either direct repression of glutathione peroxidase 4 (GPX4) or by indirect inhibition of the system Xc-, a cystineglutamate antiporter composed of Slc7a11 (system Xc-) and Slc3a2 (Yang et al., 2014; Stockwell et al., 2017). GPx4 is a unique intracellular antioxidant enzyme, which can directly reduce the phospholipid peroxide produced in the cell membrane. Slc7a11 mediates the uptake of extracellular cystine, a major precursor for glutathione (GSH) biosynthesis. It is worth noting that sorafenib, in addition to its well-known inhibitory effect on many angiogenesis related kinases (VEGFR, PDGFR, etc.), has also been reported to block the function of Slc7a11 and the production of GSH (Dixon et al., 2014). Therefore, a reasonable speculation is that genes related to ferroptosis may regulate sorafenib-induced cardiotoxicity.

Activating transcription factor 3 (ATF3) is a member of the ATF/cAMP-responsive element-binding protein (CREB) family of transcription factors, which is characterized by containing the basic region-leucine zipper (b-ZIP) DNA binding domain (Zhao et al., 2016). A large body of evidence suggests that the ATF3 gene is activated by a variety of cellular stress signals in many tissues, including DNA damage, oxidative stress, and cell injury (Hai et al., 1999; Yuan et al., 2013; Wang et al., 2016; Kaneko et al., 2017). Previous studies revealed important roles of ATF3 in promoting DNA damage response and maintaining genomic stability, and ATF3 can activate p53 to regulate its target genes (Wang et al., 2018; Wei et al., 2019). Notably, a recent study confirmed that ATF3 promotes erastin-induced ferroptosis by inhibiting system Xc^–^in HT1080 and RPE cells (Wang et al., 2020). Therefore, we sought to investigate whether ATF3 contributes to ferroptosis in sorafenib-induced cardiotoxicity.

In the current study, we elucidated that iron-dependent ferroptosis, rather than other known forms of programmed cell death, has a vital role in sorafenib-induced cardiotoxicity. We established the sorafenib-induced cardiac dysfunction model with C57BL/6 mice, which mimics the doses used in clinical practice. Mechanistically, down-regulation of Slc7a11, thereby inhibits the activity of GPX4, causes lipid peroxidation accumulates and triggers ferroptosis. By comparing data from the GEO database, we found that the expression of ATF3 was significantly increased in sorafenib treated human cardiomyocytes. In addition, we demonstrated that ATF3 suppressed Slc7a11 expression and promoted ferroptosis. In conclusion, our results not only provide new insights into the molecular mechanisms of ferroptosis in the process of sorafenib-induced cardiotoxicity but also highlight new therapeutic opportunities for the clinical practice.

## 2 Materials and methods

### 2.1 Chemicals and reagents

Sorafenib (#HY-10201) and Ferrostatin-1 (#HY-100579) were purchased from MedChemExpress (New Jersey, United States).

### 2.2 Animal studies

All procedures conformed to local and US National Institutes of Health guidelines, including the US National Institutes of Health Guide for Care and Use of Laboratory Animals, and were approved by the Ethics Committee of the Second Affiliated Hospital of Harbin Medical University (Protocol No. Sydwgzr 2020-220).

Male C57BL/6 mice (age, 6 weeks) were purchased from Beijing Weitong Lihua Experimental Animal Technology Co. Ltd (Beijing, China). After adaptively feeding for 1 week, mice were randomly divided into three groups (*n* = 12/group): 1) 10% DMSO+40% PEG300 + 5% Tween-80 + 45% saline (Control); 2) Sorafenib at 30 mg/kg/day through intraperitoneal administration (i.p); 3) Sorafenib/Fer-1 in combination: the mice were given Fer-1 (1 mg/kg/day) by intraperitoneal injection for 1 day before sorafenib injection. 14 days after sorafenib injection, cardiac function was evaluated by transthoracic echocardiography. Subsequently, animals were euthanized to harvest hearts after anaesthesia with an overdose of isoflurane, and blood was collected *via* retro-orbital sinus for further detection (Lindsey et al., 2018). Cardiac tissues were snap frozen in liquid nitrogen and kept at −80°C later or fixed with 4% paraformaldehyde for histological analysis. Experiments were carried out in accordance with ARRIVE guidelines.

### 2.3 Echocardiography

Two-dimensional-guided M-mode echocardiography examination was conducted using a ML6-15-D Linear Probe (GE, Vivid E9, United States). Mice were anesthetized with light anesthesia (1–2% isoflurane) and then the functional parameters were recorded in a blinded manner (Lindsey et al., 2018). The percentage of ejection fraction (%EF), fraction shortening (%FS) and other cardiac functions were calculated by the built-in software package.

### 2.4 Histology staining

After fixation, the hearts were embedded in paraffin, cut into 2-μm sections, and stained with hematoxylin eosin (H&E) (Solarbio, G1121, China) and Masson’s trichrome staining (Solarbio, G1346, China). Cardiomyocyte vacuolization was detected by H&E staining, while myocardial fibrosis was assessed upon Masson’s staining. Images of the staining were taken using a light microscope and ImageJ software was used to evaluate collagen deposition.

### 2.5 Transmission electron microscopy

Tissue and cells were fixed in 2.5% glutaraldehyde and then overnight at 4°C. Samples were rinsed in PBS, postfixed in 1% osmium in PBS for 2 h, dehydrated in a graded series of ethanol and placed in isoamyl acetate. Finally, the Epon812 epoxy resin embedding agent was polymerized at −80°C overnight. Ultrathin (70 nm) sections were cut with an ultramicrotome and stained with uranium acetate and lead citrate. Specimens were observed under a scanning electron microscope (Hitachi TEM system).

### 2.6 Serum biomarkers of cardiac injury

Enzyme linked immunosorbent assay (ELISA) kits were used to determinate the contents of creatine kinase isoenzyme (CK-MB) (Jingkang, JLC3886, China) and lactate dehydrogenase (LDH) (Jingkang, JLC2927, China) in mice serum.

### 2.7 MDA concentration

To evaluate levels of lipid peroxidation, malondialdehyde (MDA) was detected using an MDA assay kit (Beyotime, S0131S, China). Assays were performed according to the manufacturer’s instructions.

### 2.8 H9c2 cell culture and treatment

The H9c2 rat cardiomyoblast cell line H9c2 (CRL-1446) was purchased from the American Type Culture Collection (Rockville, MD, United States). Cells were incubated under normal cell culture conditions (37°C, 5% CO_2_) in Dulbecco’s Modified Eagle Medium (DMEM, GIBCO, 11965092, United States) supplemented with 10% fetal bovine serum (FBS, GIBCO, 16140071, United States). Cells were passed regularly and sub-cultured to 80% confluence before the cell experiment. For 12 h prior to each experiment, the cell medium was supplemented with 0.5% FBS. For *in vitro* experiments, sorafenib was added for the indicated times and concentrations. Ferrostatin-1 (15 μM) was administered 1 h before sorafenib treatment. All *in vitro* experiments were performed in triplicates per run and in three individual experiments.

### 2.9 Detection of ROS

Intracellular Reactive Oxygen Species (ROS) levels were evaluated by reactive oxygen species assay kit (Beyotime, S0033S, China). After various treatments, cells were incubated with 10 μmol/L (final concentration) DCFH-DA for 20 min at 37°C and washed afterwards. Fluorescence images were acquired blinded to condition using a fluorescence microscope.

Lipid oxidation was detected via BODIPY 581/591 C11 (D3861, ThermoFisher Scientific) following manufacturers’ instructions. Images were obtained using the Leica DMi8 Inverted Microscope.

### 2.10 JC-1 staining

Mitochondrial membrane potential (ΔΨm) was detected using the JC-1 dye (Beyotime, C2006, China). In brief, the cells were incubated with JC-1 for 20 min at 37°C. Then, the cells were washed again with PBS, and the images for JC monomers (Green fluorescence; 514/529 nm) and JC aggregates (Red fluorescence; 585/590 nm) were photographed by a fluorescence microscope (DMi8; Leica, Microsystems, Germany). ΔΨm is expressed as a relative aggregate-to-monomer (red/green) fluorescence intensity ratio.

### 2.11 Western blotting

Total protein was extracted with RIPA buffer and the protein concentration was determined by a BCA assay kit (Beyotime, P0012, China). Equal amounts of protein samples were separated by SDS-PAGE (Sevenbio, Beijing, China), and transferred onto a polyvinylidene difluoride membrane (Millipore, China). The membrane was blocked with 5% fat-free milk for 1 h at room temperature and then incubated with primary antibodies overnight at 4°C. The following antibodies were used: Slc7a11 (1:3,000) (Abcam, ab175186), GPX4 (1:3,000) (Abcam, ab125066) and GAPDH (1: 10000) (Abcam, ab181602). The corresponding horseradish peroxidase HRP-conjugated secondary antibody (1:5,000) (Abcam, ab6721) was incubated at room temperature for 1 h. Immunoreactive bands were developed with the chemiluminescence ECL detection system (Tanon 5,100, China). Densitometry analysis was performed using ImageJ software. Protein expression levels were normalized to the levels of GAPDH, which was used as an internal control.

### 2.12 RealTime-qPCR

Total RNA was extracted using TRIzol reagent (Invitrogen, 15596026, United States), and cDNA was synthesized with Transcriptor First Strand cDNA Synthesis kit (Roche, 04896866001, Germany). Quantitative real-time PCR was performed using SYBR Green I Master (Roche, 4707516001, Germany), and triplicate samples were run on a CFX96 Touch Real-Time PCR Detection System according to the manufacturer’s protocol. Normalised gene expression values (against GAPDH) were obtained using the ΔΔCT method. All details of the PCR primer sequences are presented in Supplemental Table S1.

### 2.13 siRNA plasmid and transfection

Short interfering RNAs (siRNAs) and overexpression plasmids of Slc7a11, ATF3 and the negative control were designed and synthesized by GenePharma (Shanghai, China), and transfected into H9c2 cells using Lipofectamine 3,000 transfection reagent (Invitrogen, L3000015, United States) according to the manufacturer’s instructions. The corresponding siRNA sequence is presented in Supplemental Table S2.

### 2.14 Data collection

The gene expression profile of GSE146096 was obtained from the Gene Expression Omnibus (GEO) database (http://www.ncbi.nlm.nih.gov/geo/) based on the platform of Illumina HiSeq 2,500 (*Homo sapiens*). The data set was last updated on 13 October 2020. Totally, four sorafenib treated adult human cardiomyocyte lines were obtained. There were 13 control samples and 4 sorafenib treated samples in line A, 8 control samples and 4 sorafenib treated samples in line B, 7 control samples and 3 sorafenib treated samples in line D, 11 control samples and 3 sorafenib treated samples in line E, respectively. We downloaded the 259 FRGs data from the FerrDb web portal (http://www.zhounan.org/ferrdb).

### 2.15 Bioinformatics analysis

The mRNAs in the data matrix were extracted and analyzed using R software (V.3.6.3). Log-fold change (FC) and adjusted *p*-values (adj. *P*) were calculated relative to control samples. The false positive correction of adj. *P* for differential abundance was performed using the Benjamini–Hochberg correction. Adj. *p* < 0.05 and |logFC|>1.0 was set as the specifc cut-of criteria of differentially expressed genes (DEGs). Differential expression analysis was conducted by using the “DESeq2” R package. The Venn diagram were drawn using the “ggplot2” R package.

### 2.16 Statistics

All data was presented as mean ± standard deviation (SD) and analyzed using GraphPad Prism software (version 8). To determine statistical significance, unpaired Student’s t-test and one-way analysis of variance (ANOVA) were used. If ANOVA revealed a significant difference, pairwise comparisons between group means were performed with Tukey post hoc test. A *p* value less than 0.05 was considered statistically significant.

## 3 Results

### 3.1 Ferrostatin-1 prevented sorafenib-induced cardiac dysfunction and death

**FIGURE 1:**
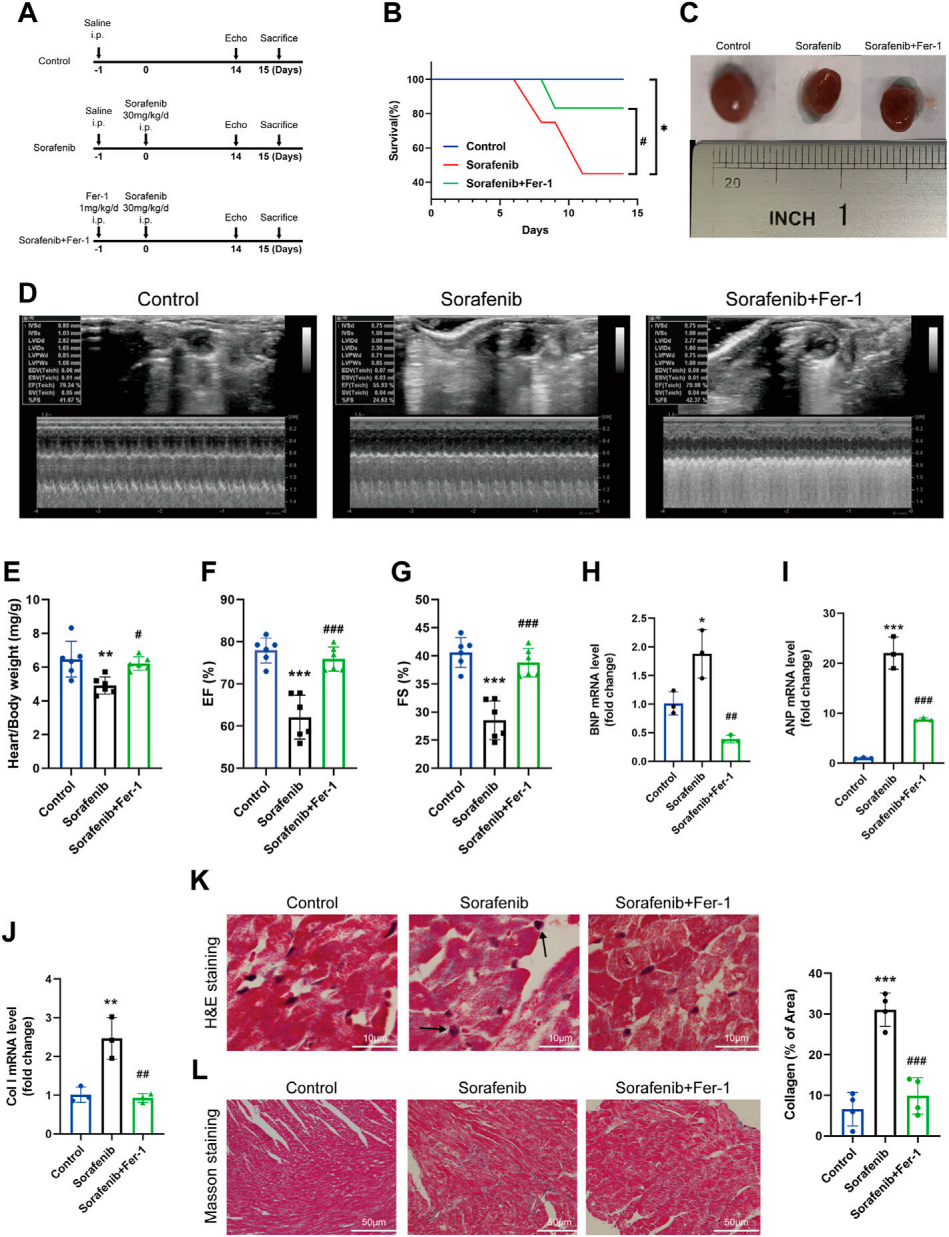
Fer-1 reduces sorafenib-induced heart dysfunction in mice. **(A)** Schematics of the overall design outline. **(B)** Kaplan Meier plot of survival. **(C)** Representative images of the heart in different groups. **(D)** Representative M-mode echocardiographic images of each study group. **(E)** Heart weight to body weight ratio (*n* = 6 per group) **(F)** EF, ejection fraction (*n* = 6 per group). **(G)** FS, fractional shortening (*n* = 6 per group). **(H–J)** Levels of col I, BNP and ANP in heart tissue (*n* = 3 per group). **(K)** The myocardial pathological damage was detected by HE staining. Scale bar, 10 μm. **(L)** Masson’s trichrome staining was performed on the cardiac sections for collagen fibers. Scale bar, 50 μm *p < 0.05, **p < 0.01, ***p < 0.001 vs. control group. #*p* < 0:05, ##*p* < 0.01, ###*p* < 0.001 vs. sorafenib group. One-way analysis of variance (ANOVA) with Tukey’s post hoc test was used for multigroup comparisons.

To establish the sorafenib-induced cardiac injury model, C57BL6 mice were injected intraperitoneally with the indicated treatment, which mimics the doses used in clinical practice (Figure 1A) (Wilhelm et al., 2004). The survival rate was measured for 14 days (Figure 1B). Immediately after killing, the body and heart weight were determined and expressed as a heart/body weight ratio. As expected, sorafenib treatment resulted in more mice death than the control group. Sorafenib-treated mice show a decreased heart-to-body weight ratio compared with the vehicle group (Figures 1C,E). Cardiac function was monitored by transthoracic echocardiography before euthanasia. Sorafenib caused a significant loss in cardiac contractile function, reflected by reduced EF% and FS% and each of which was significantly improved by Fer-1 (Figures 1D,F,G). These data indicated that sorafenib induced cardiac injury in mice reflected by the systolic function of left ventricular and increased mortality.

Hematoxylin-eosin (HE) staining further validated sorafenib-induced heart injury. As shown in Figure 1H, sorafenib treatment caused disordered myocardium and enlarged cardiomyocytes, and marked nuclear fragmentation was apparent, suggesting the mechanism of myocardium necrosis. Fer-1 pretreatment significantly reduced the degree of myocardial injury (Figure 1K). Masson’s trichrome staining was performed to see the collagen deposition and fibrosis in the heart tissue. Following intraperitoneal administration of sorafenib, we found that the collagen content and fibrosis were increased in mouse heart tissues (Figure 1L). Additionally, when the expression mRNA levels of Col I, ANP and BNP in the heart were significantly higher than in control mice. However, Fer-1 administration attenuated these increases significantly (Figures 1H–J). Altogether, these findings suggest that Fer-1 prevents sorafenib-induced cardiac fibrosis and myocardial damage.

### 3.2 Ferroptosis was triggered in sorafenib-induced cardiotoxicity

**FIGURE 2:**
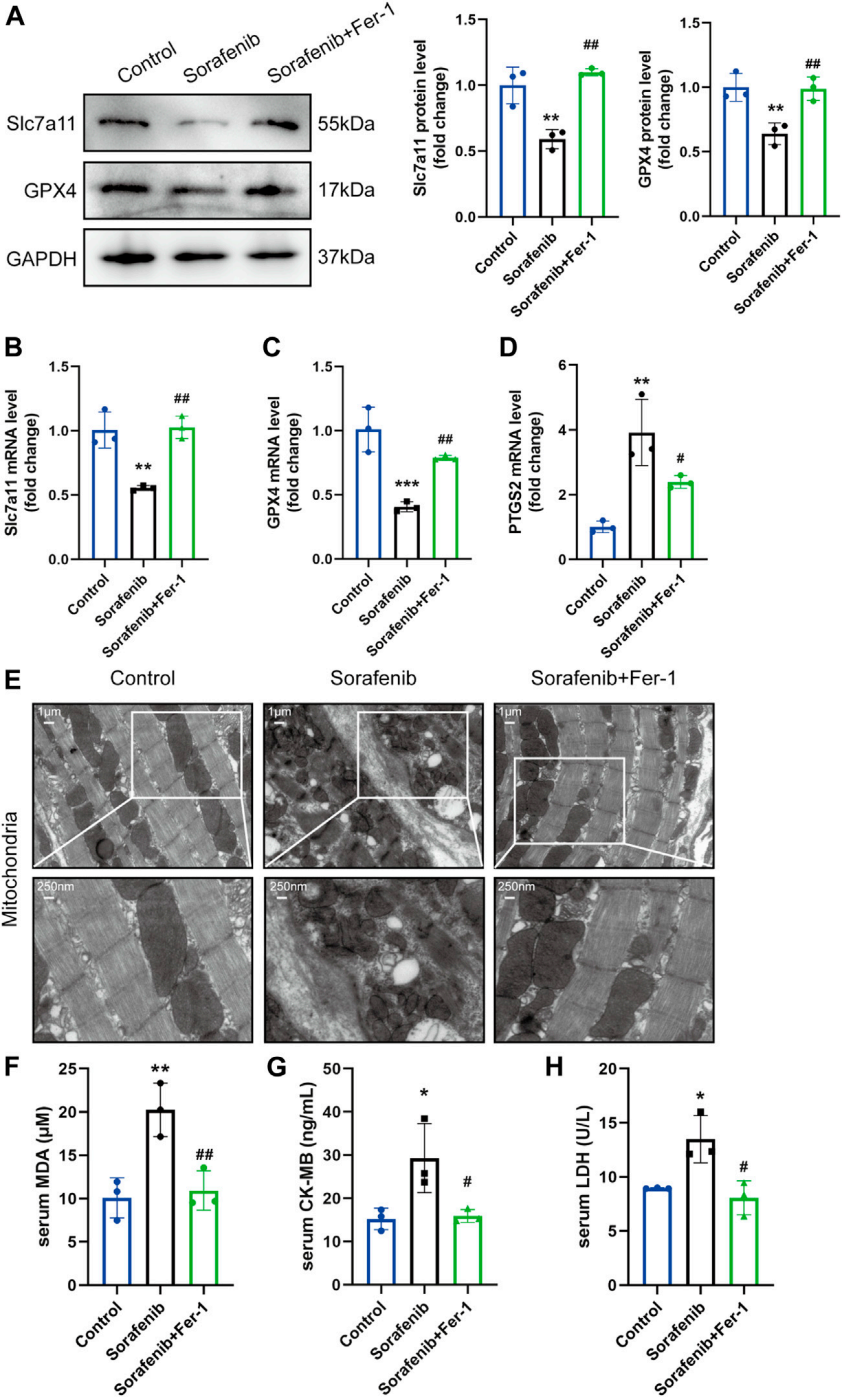
Ferroptosis is activated in mice injected with sorafenib. **(A)** Representative western blots of Slc7a11 and GPX4 are shown with GAPDH used as a loading control (*n* = 3 per group). **(B–D)** The mRNA level of Slc7a11, GPX4 and PTGS2 (*n* = 3 per group). Data are represented as means ± SD for three biological replicates. **(E)** Representative images of SEM (Whole image scale bar = 1 μm, zoomed sections = 250 nm). **(F–H)** The serum MDA, CK-MB, LDH levels in indicated groups (*n* = 3 per group). *p < 0.05, **p < 0.01 vs. control group. #*p* < 0:05, ##*p* < 0.01 vs. sorafenib group. One-way ANOVA with Tukey’s post hoc test was used for multigroup comparisons.

In order to explore the impact of ferroptosis in sorafenib induced cardiac injury, we further measured the expression levels of Slc7a11 and GPX4 in the heart, which are core targets of ferroptosis, thus reducing phospholipid hydroperoxides using GSH and preventing ferroptotic cell death. Both Slc7a11 and GPX4 were decreased in the sorafenib group compared to the vehicle group, and both were increased in Fer-1 administration compared with the sorafenib group (Figures 2A–C). More importantly, the ferroptosis marker PTGS2 was significantly upregulated after sorafenib treatment, showing that myocardial cells undergo a ferroptosis process *in vivo* as well (Figure 2D). To confirm the existence of ferroptosis, we further utilized scanning transmission electron microscopy to detect the ultrastructural changes of cardiomyocytes. Sorafenib caused severe myofilament disassembly or disorganized myofilaments, while these pathological changes can be alleviated by Fer-1 treatment (Figure 2E). Meanwhile, biochemical analysis revealed that serum CK-MB and LDH, clinical biomarkers of myocardial injury, were significantly elevated by sorafenib treatment (Figures 2G,H). The MDA level in the serum was also increased (Figure 2F). However, Fer-1 administration attenuated these increases significantly. Taken together, these data suggest that ferroptosis was activated in sorafenib-induced cardiotoxicity.

### 3.3 Ferroptosis was activated in H9c2 cardiomyoblasts by sorafenib

**FIGURE 3:**
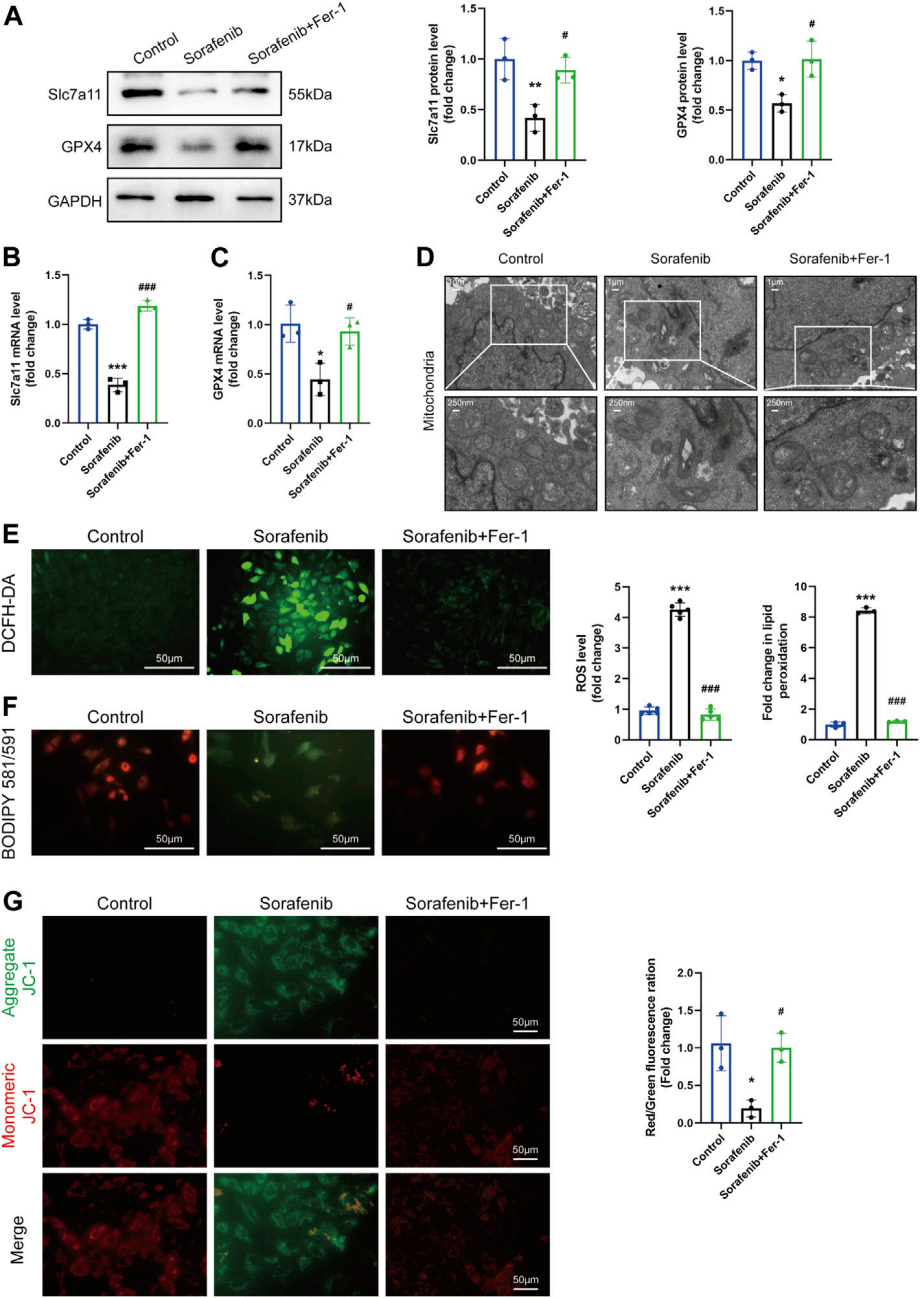
Ferroptosis in H9c2 cardiomyoblasts is activated after sorafenib treatment. **(A–C)** The mRNA and protein level of Slc7a11 and GPX4. Data are represented as means ± SD for three biological replicates. **(D)** Representative transmission electron microscopy images (Whole image scale bar = 1 μm, zoomed sections = 250 nm). **(E)** Intracellular ROS levels were evaluated by DCFH-DA staining. Scale bar = 50 μm. **(F)** The generation of lipid ROS was determined by the C11-BODIPY probe. Scale bar = 50 μm. **(G)** JC-1 aggregates show red fluorescence, indicating high mitochondrial membrane potential, and JC-1 monomers show green fluorescence. Scale bar = 50 μm *p < 0.05, **p < 0.01, ***p < 0.001 vs. control group. #*p* < 0:05, ###*p* < 0.001 vs. sorafenib group. One-way ANOVA with Tukey’s post hoc test was used for multigroup comparisons.

We tested various time points and doses of sorafenib to identify its optimized time and concentration. After 48 h of treatment, it was found that the expression level of Slc7a11 and GPX4 was efficiently decreased at a concentration of 5 μM (Supplementary Material online, Supplementary Figure S1). Meanwhile, the expression of Slc7a11 and GPX4 mRNA and protein were verified with or without Fer-1 (Figures 3A–C). Ultrastructural analysis by transmission electron microscopy revealed that 5 µM sorafenib exposure produced mitochondrial swelling, cristae disorientation and breakage in H9c2 cells (Figure 3D). However, the derangement in the ultrastructural morphology of mitochondria was improved in the Fer-1 cotreatment group, as evidenced by normalized crista density and architecture (Figure 3D). The production of ROS, which promotes mitochondrial oxidative stress, plays a crucial role in the development of ferroptosis. Sorafenib treatment caused a significant upregulation of intracellular ROS, lipid ROS and loss of ΔΨm, but Fer-1 cotreatment reduced this sorafenib-induced effect, presumably by decreasing ROS level and maintaining mitochondrial ΔΨm (Figures 3E–G).

### 3.4 Knock-down of Slc7a11 sensitized cardiomyoblasts to sorafenib-induced ferroptosis

**FIGURE 4:**
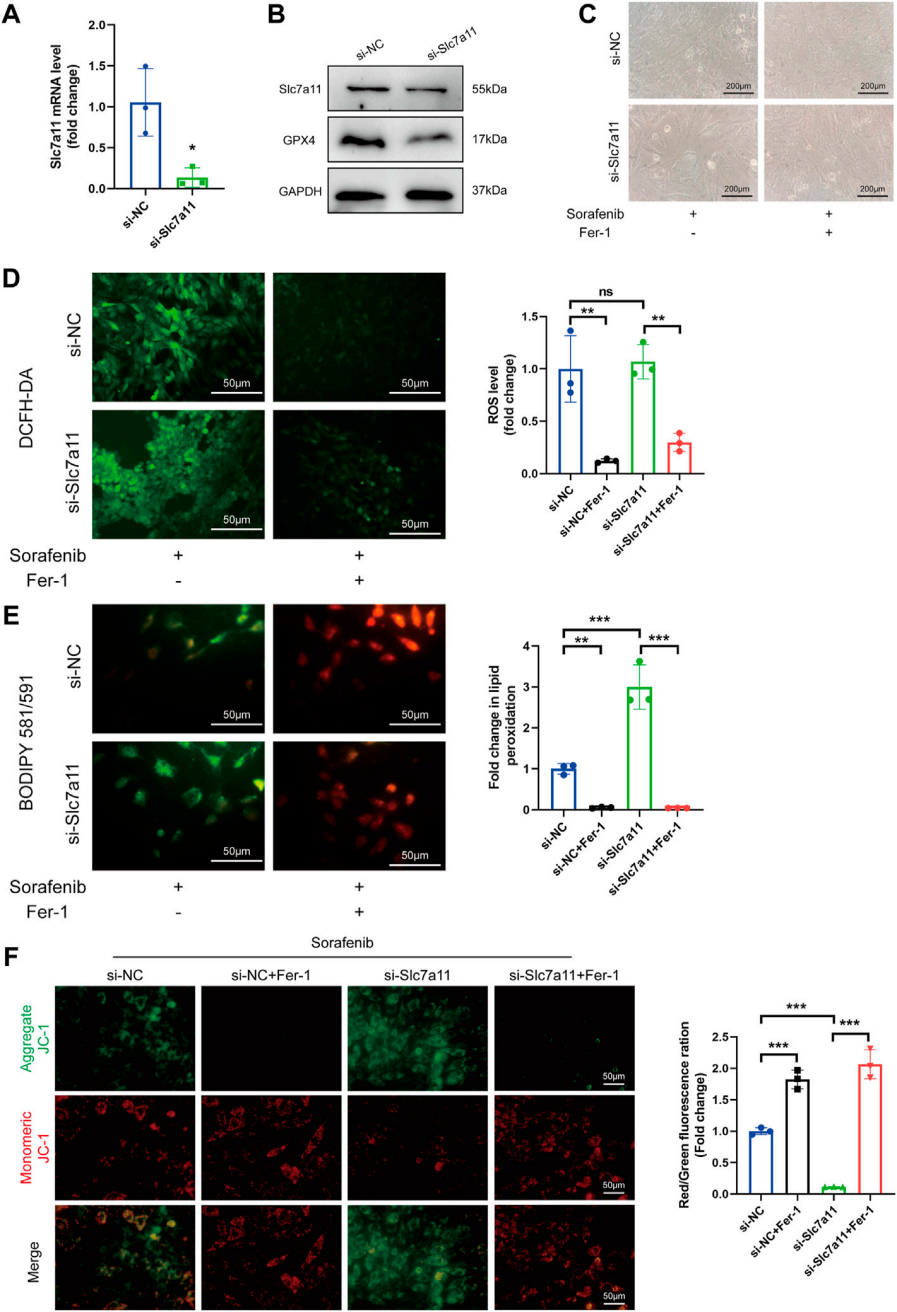
Knock-down of Slc7a11 sensitizes H9c2 cells to sorafenib-induced cardiac ferroptosis. **(A)** Relative expression level to si-NC is shown. Data are represented as means ± SD for three biological replicates. *p < 0.05 vs. si-NC group by Student’s t test. **(B)** The expression of Slc7a11 and GPX4 was determined by Western blot analysis. **(C)** Cellular morphology under a phase contrast microscope. Scale bar = 200 μm. **(D)** Representative images and ROS level. Scale bar = 50 μm. **(E)** The generation of lipid ROS was determined by the C11-BODIPY probe. Scale bar = 50 μm. **(F)** JC-1 staining for mitochondrial membrane potential. Scale bar = 50 μm **p < 0.01, ***p < 0.001 vs. indicated group. ns, no significance. One-way ANOVA with Tukey’s post hoc test was used for multigroup comparisons.

Considering the significant decrease in Slc7a11 expression by sorafenib shown in our study, we hypothesized that Slc7a11 may have the potential capacity to inhibit ferroptosis. To corroborate these findings, siRNA was used to knock down Slc7a11 in H9c2 cardiomyoblasts. Compared with the control group, Slc7a11 was reduced in the Slc7a11-knocked down group (Figures 4A,B). Also, in the Slc7a11 knocked-down group, GPX4 was reduced as compared to the control group (Figure 4B). In addition, Slc7a11 deficiency aggravated oxidative stress. However, Fer-1 alleviated the intracellular ROS and lipid ROS levels caused by sorafenib and Slc7a11 knockdown (Figures 4C–E). JC-1 staining also indicated that Slc7a11 deficiency aggravated sorafenib induced JC-1 monomers, whereas Fer-1 co-treatment inhibited the tendency (Figure 4F). These data suggest that knockdown of Slc7a11 in cardiomyoblasts enhances the sensitivity to sorafenib toxicity.

### 3.5 Slc7a11 overexpression suppressed ferroptosis and sorafenib cytotoxicity

**FIGURE 5:**
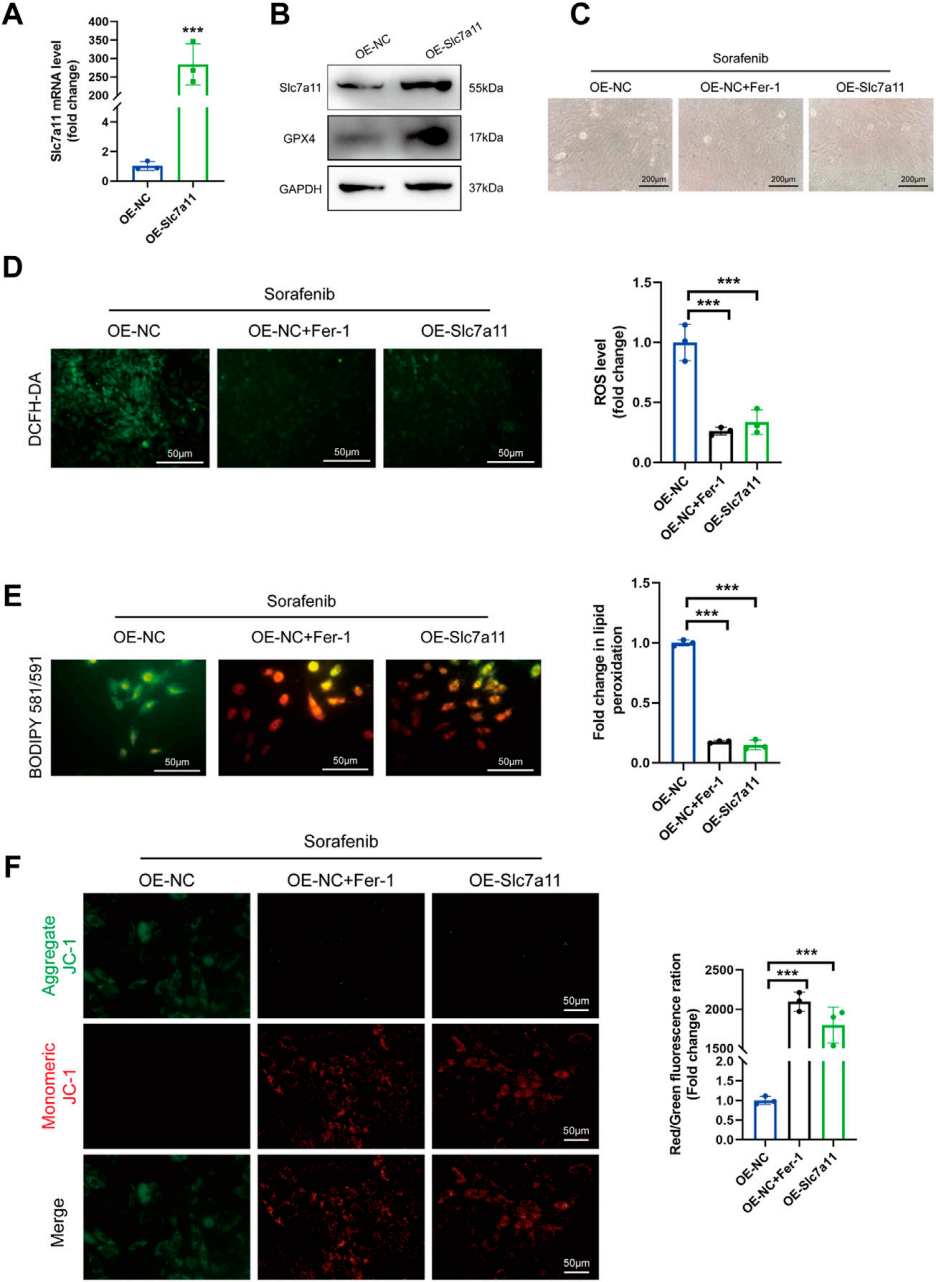
Slc7a11 overexpression suppressed ferroptosis and sorafenib cytotoxicity. **(A)** Relative mRNA levels of Slc7a11 in cultured H9c2 cells. Data are represented as means ± SD for three biological replicates. ***p < 0.001 vs. OE-NC group by Student’s t test. **(B)** Representative western blot results of Slc7a11 and GPX4. **(C)** Cellular morphology (Scale bar = 200 μm), **(D)** intracellular ROS level, **(E)** Intracellular lipid ROS and **(F)** ΔΨm are shown. Scale bar = 50 μm ***p < 0.001 vs. indicated group. One-way ANOVA with Tukey’s post hoc test was used for multigroup comparisons.

Slc7a11 overexpression efficiency was validated by qRT-PCR and Immunoblotting (Figures 5A,B). We also found that the protein expression of GPX4 was increased after Slc7a11 overexpression (Figure 5B). The results showed that sorafenib led to the accumulation of enhanced accumulation of ROS, resulting in the depolarization of the mitochondrial membrane in H9c2 cells (Figures 5C–F). In contrast, Slc7a11 overexpression significantly diminished ROS accumulation and preserved ΔΨm (Figures 5D–F). Taken together, these findings demonstrate that Slc7a11 plays an important protective role in the process of sorafenib-induced ferroptosis.

### 3.6 The identification of ferroptosis DEGs involved in sorafenib-induced cardiotoxicity

**FIGURE 6:**
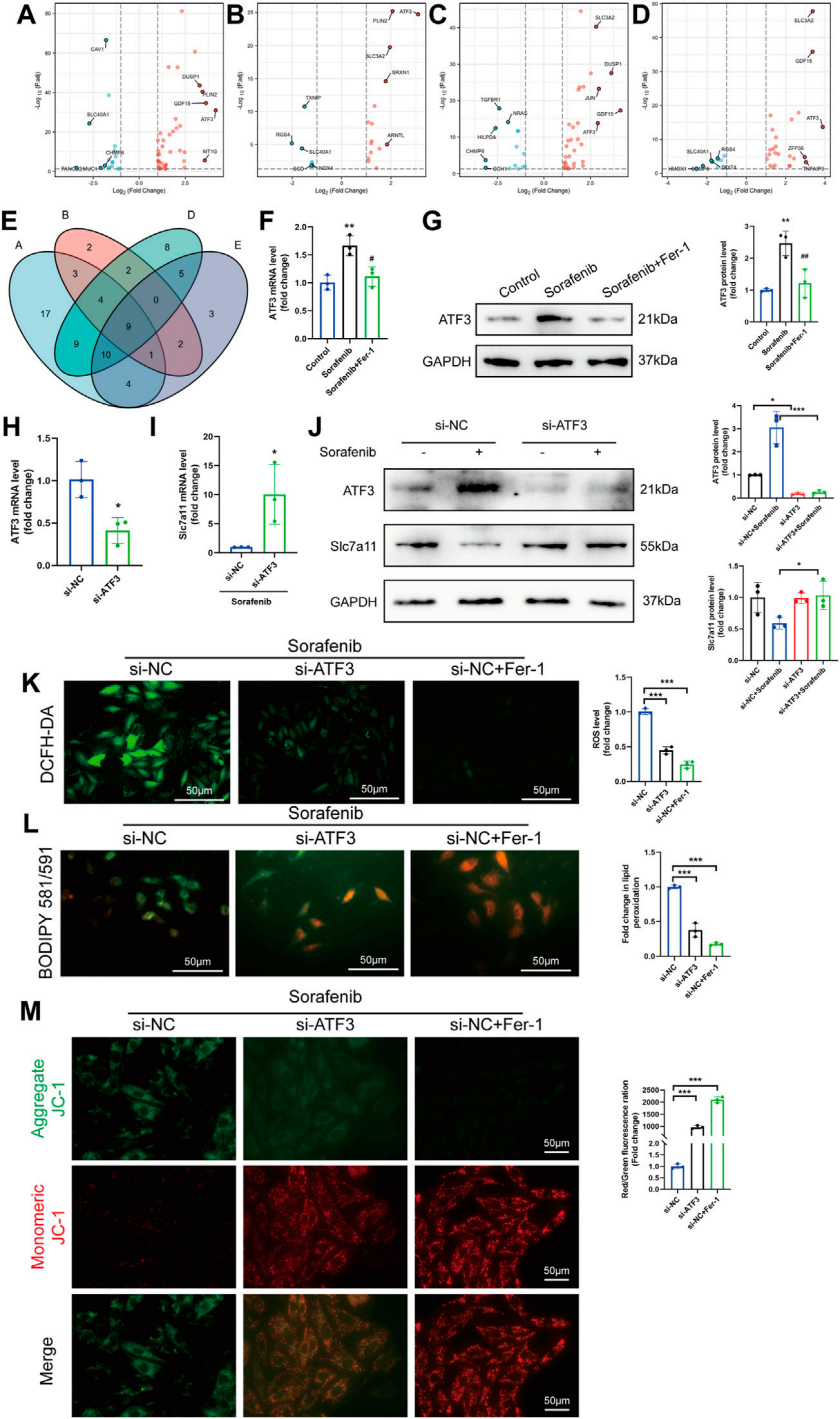
The identification of ferroptosis DEGs involved in sorafenib-induced cardiotoxicity. **(A–D)** Volcano plot of DEFRGs in line A, line B, line D and line E. **(E)** Interactions between DEFRGs in line A, line B, line D and line E **(F–G)** Relative mRNA and protein levels of ATF3 in cultured H9c2 cells. Data are represented as means ± SD for three biological replicates. **p < 0.01 vs. control group. #*p* < 0:05, ##*p* < 0.01 vs. sorafenib group. One-way ANOVA with Tukey’s post hoc test was used for multigroup comparisons. **(H–I)** The mRNA expression of ATF3 and Slc7a11 after knockdown of ATF3 in H9c2 cells were analyzed by qRT-PCR. Data are represented as means ± SD for three biological replicates. *p < 0.05 vs. si-NC group by Student’s t test. **(J)** Protein expression of ATF3 and Slc7a11 were analyzed with or without sorafenib or/and siRNA in H9c2 cell lines. **(K–M)** H9c2 cells were treated with sorafenib after being transduced with siRNAs or Fer-1 **(K)** Intracellular ROS level, **(L)** Intracellular lipid ROS and **(M)** ΔΨm are shown. Scale bar = 50 μm *p < 0.05, ***p < 0.001 vs. indicated group. One-way ANOVA with Tukey’s post hoc test was used for multigroup comparisons.

**FIGURE 7:**
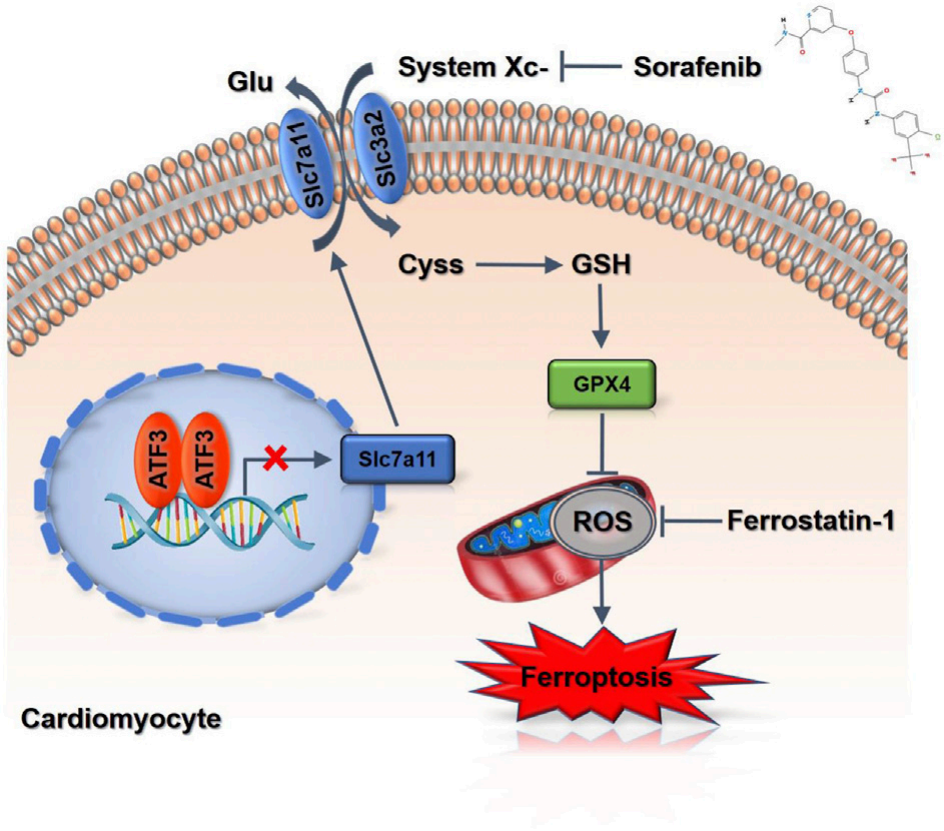
A scheme of the proposed mechanisms. Sorafenib treatment increases ATF3 expression by repressing Slc7a11 expression to suppress system Xc-in cardiomyocytes, and thereby predispose cells to a state prone to ferroptosis.

The study flow-chart was developed in Supplementary Material online, Supplementary Figure S2. To study the transcriptomic response to sorafenib associated with cardiotoxicity, we obtained the mRNA expression data of sorafenib treated four different adult human cardiomyocytes from the GEO database (GSE146096) (Supplementary Material online, Supplementary Tables S3–S6). We also gained the dataset including 259 genes from the Ferroptosis Database (FerrDb) and intersected them with GSE146096 to identify ferroptosis DEGs (Supplementary Material online, Supplementary Table S7). The volcano plot displayed the differentially expressed ferroptosis-related genes (DEFRGs) between the sorafenib treated and normal samples in the four primary cardiomyocyte lines (Figures 6A–D, 7D). The Venn diagram revealed that 9 DEFRGs intersected between 4 cell lines (Figure 6E). Relevant details of all gene lists and overlap are available in Table 1.

**TABLE 1:** Summary of the Co-Expressed DEFRGs in 4 cell lines.

Gene	Line A		Line B		Line D		Line E	
	log2FC	adj. *P*	log2FC	adj. *P*	log2FC	adj. *P*	log2FC	adj. *P*
ATF3	4.153643	1.09E-31	3.0964314	1.91E-25	2.90307	1.41E-14	3.88816	2.20E-14
GDF15	3.624228	2.02E-35	1.3333789	1.46E-11	4.122479	5.09E-18	3.36879	1.39E-36
PLIN2	3.437316	3.59E-41	2.0654185	6.16E-26	2.129018	1.60E-24	2.65806	1.27E-18
SLC3A2	3.027467	2.08E-61	1.9550553	1.73E-20	2.813905	5.39E-41	3.37827	1.63E-48
DDIT3	2.463543	1.64E-10	1.2289242	7.30E-09	2.530736	3.12E-28	2.306	1.19E-16
TRIB3	2.2243	8.33E-24	1.5357126	4.26E-05	2.021348	9.49E-09	2.424	4.66E-05
SRXN1	2.026973	1.05E-22	1.7868464	2.55E-15	1.623878	0.00169	1.3531	0.01670976
CHMP6	−1.86705	0.001051	−1.187848	0.007537	−3.14597	0.000207	−2.207	0.00653744
SLC40A1	−2.70885	4.71E-25	−1.601869	3.95E-05	−1.47528	4.20E-08	1.77905	0.00016727

FC, fold change. DEFRGs, differentially expressed ferroptosis-related genes.

### 3.7 ATF3 might promote sorafenib-induced ferroptosis by system Xc^−^ inhibition

Among the obtained DEFRGs, ATF3, which was the most highly differentially expressed gene, caught our attention. A recent finding that the ATF3 promotes erastin-induced ferroptosis by system Xc− inhibition prompted us to speculate that ATF3 may play an important role in the process of ferroptosis (Wang et al., 2020). Indeed, ROS-mediated ATF3 superinduction required nuclear translocation of the ROS-sensitive transcription factor Nrf2 after binding to the ATF3 promoter (Okamoto et al., 2006; Kim et al., 2010). We further confirmed the effect of sorafenib or Fer-1 on the expression of ATF3 level. Sorafenib increased the ATF3 expression, which was then significantly reduced by Fer-1 administration (Figures 6F,G). Since the expression level of Slc7a11 was positively correlated with system Xc^−^ activity, we detected whether knock-down of ATF3 activated system Xc^−^ activity by up regulating the expression of Slc7a11 (Lewerenz et al., 2013). The mRNA and protein levels of ATF3 were reduced by ATF3 knock-down in cardiomyoblasts (Figures 6H,J). In addition, sorafenib fails to suppress Slc7a11 expression in ATF3-knockdown cells (Figures 6I,J). As expected, ATF3 deficiency significantly diminished the intracellular ROS and lipid ROS accumulation and preserved ΔΨm (Figures 6K–M). Taken together, we demonstrated that ATF3 promotes ferroptosis in sorafenib-induced cardiotoxicity by suppressing Slc7a11.

## 4 Discussion

In the current study, we elucidated that iron-dependent ferroptosis, rather than other known forms of programmed cell death, has a vital role in sorafenib-induced cardiotoxicity. We established the sorafenib-induced cardiac dysfunction model with C57BL/6 mice, which mimics the doses used in clinical practice. Mechanistically, down-regulation of Slc7a11, thereby inhibits the activity of GPX4, causes lipid peroxidation to accumulate and triggers ferroptosis. By comparing data from the GEO database, we found that the expression of ATF3 was significantly increased in sorafenib treated human cardiomyocytes. In addition, we demonstrated that ATF3 suppressed Slc7a11 expression and promoted ferroptosis. In conclusion, our results not only provide new insights into the molecular mechanisms of ferroptosis in the process of sorafenib-induced cardiotoxicity but also highlight new therapeutic opportunities for the clinical practice.

Ferroptosis is a non-apoptotic mode of cell death that depends on production of lethal levels of iron-dependent lipid ROS (Dixon et al., 2012; Cao and Dixon, 2016; Xie et al., 2016; Yang and Stockwell, 2016; Stockwell et al., 2017). Recent evidence has suggested that ferroptosis plays an important role in the pathogenesis of cardiotoxicity. In the study by Fang et al., ferroptosis involved in doxorubicin-induced cardiac injury in a heme oxygenase-1 (hmox1)-dependent manner was found. Hmox1 is responsible for the degradation of heme to free iron, which ultimately leads to ROS accumulation, particularly in the mitochondria, resulting in lipid peroxidation. Combined with ferroptosis inhibitors, iron chelation therapy prevents the resulting oxidative tissue damage and cardiotoxicity (Fang et al., 2019). In addition, the anti-ferroptotic protein GPX4 plays a key role in the cytosol and mitochondria in doxorubicin-induced ferroptosis and inducing excessive lipid peroxidation through doxorubicin–Fe^2+^ complex in the mitochondria. Another study revealed the role and potential mechanism of ferroptosis on lipopolysaccharide (LPS)-induced cardiac injury (Li et al., 2020). Mice were injected with LPS to generate experimental sepsis and Fer-1 were used to inhibit ferroptosis of mice with sepsis-induced cardiac injury (Li et al., 2020). These findings emphasize the significance of the ferroptosis in doxorubicin or LPS and a key role in this regard is played by mitochondria (Fang et al., 2019; Li et al., 2020; Tadokoro et al., 2020). However, there is no evidence of ferroptosis playing a role in sorafenib-induced cardiac injury.

Our study documented a reduction of cardiac function and an increasing rate of mortality in sorafenib-treated mice, with better preservation of cardiac contractile properties and a lower death rate in Fer-1 cotreatment animals. Fer-1, a potent and selective small-molecule inhibitor of ferroptosis, significantly attenuated the accumulation of lipid hydroperoxides and thereby inhibited cell death. Further, ferroptotic-like morphological alterations are observed primarily in sorafenib-treated murine hearts, which are characterized by mitochondrial outer membrane rupture and mitochondrial cristae reduction. Evidence for ferroptosis that shows increased lipid peroxidation was also confirmed by increased MDA concentration and decreased GPX4 and Slc7a11 levels, changes that are also the signature of ferroptosis. Cell death by ferroptosis is characterized by mitochondrial dysfunction and accumulation of lipid ROS.

Slc7a11 is well known to protect cells in response to oxidative stress and to maintain intracellular glutathione levels (Liu et al., 2020). Qian et al. found that the suppression of xCT activity results in intracellular cysteine depletion and impairs GSH synthesis, thereby inducing severe oxidative stress and lung injury (Qian et al., 2018). These results were consistent with our study. Results from our study by knock-down Slc7a11 or overexpression of Slc7a11 verified the beneficial role of Slc7a11 in sorafenib-induced cardiotoxicity. We evaluated lipid peroxidation and GPx4 expression levels, which are two critical parameters associated with ferroptosis. While system Xc− contributes to the maintenance of redox homeostasis and the blockage of lipid peroxidation, despite it is largely unknown how Xc− activity is regulated. Previously, Nrf2 and ATF4 were shown to bind to the Slc7a11 promoter and activate its expression, binding of p53 to the Slc7a11 promoter represses Slc7a11 transcription (Ye et al., 2014; Jiang et al., 2015; Chen et al., 2017). Recently, an *in vitro* study identified ATF3 as a major repressor of Slc7a11. ATF3 binds to the Slc7a11 promoter, inhibits its transcription and promoted ferroptosis induced by erastin (Wang et al., 2020). Also, bioinformatic analysis of our study substantiated the role of ATF3 in transcriptional regulation. We analyzed the GEO databases and found that sorafenib induces ATF3 gene expression in human cardiomyocytes. In addition, we demonstrated that ATF3 suppressed Slc7a11 expression and promoted ferroptosis. Altogether, this study provides insights into mechanisms underlying ferroptosis in the process of sorafenib-induced cardiotoxicity and targeting ATF3 may provide feasible therapies for sorafenib-induced cardiotoxicity.

## Data Availability

The datasets presented in this study can be found in online repositories. The names of the repository/repositories and accession number(s) can be found in the article/Supplementary Material.
