# Supplementary figures and images for: ATF3 promotes ferroptosis in sorafenib-induced cardiotoxicity by suppressing Slc7a11 expression
Source: Front Pharmacol. 2022 Sep 23;13:904314. doi: 10.3389/fphar.2022.904314 (PMC9537618; doi:10.3389/fphar.2022.904314)

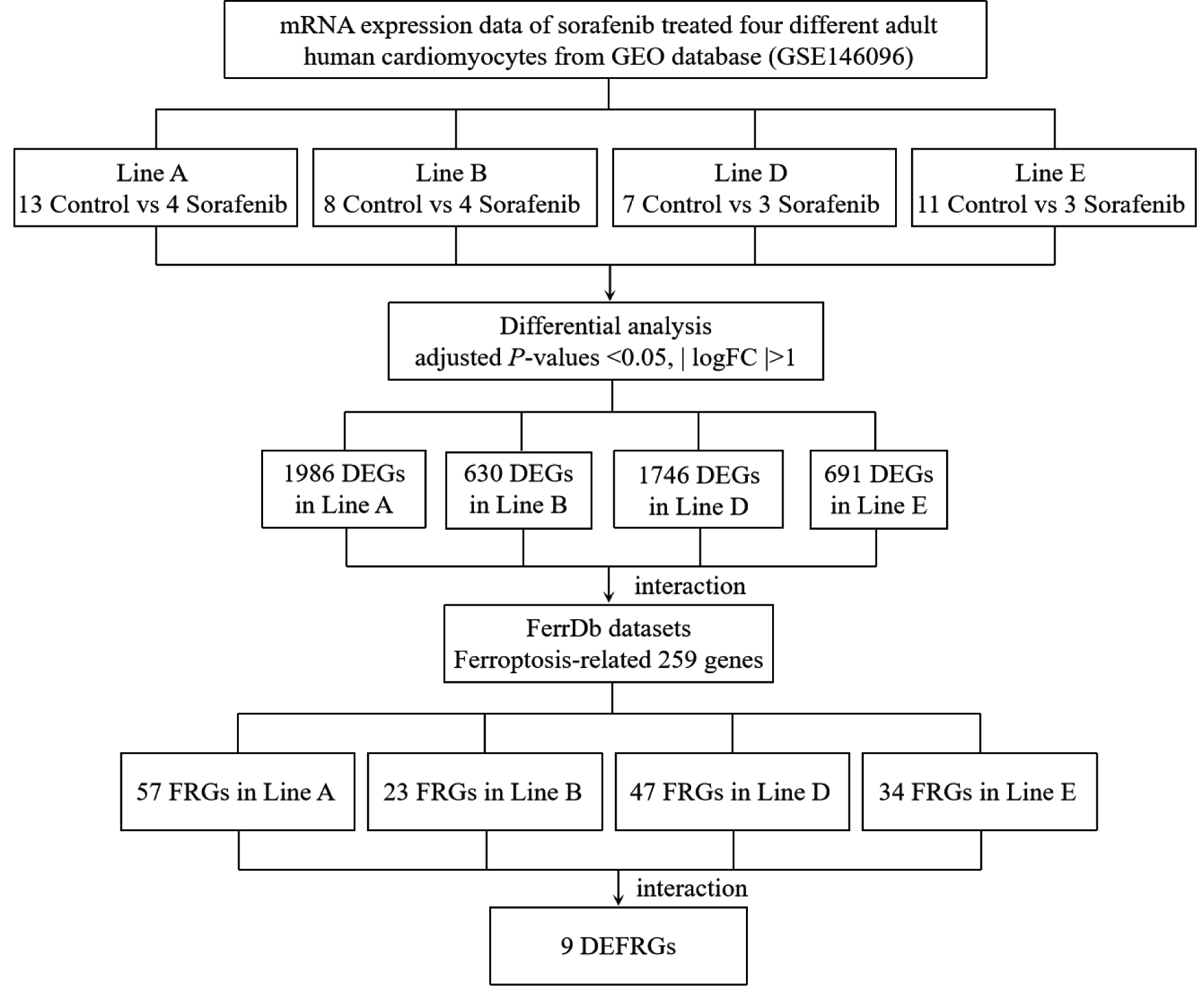

Supplement: Supplementary file 3 [file Image2.TIF]

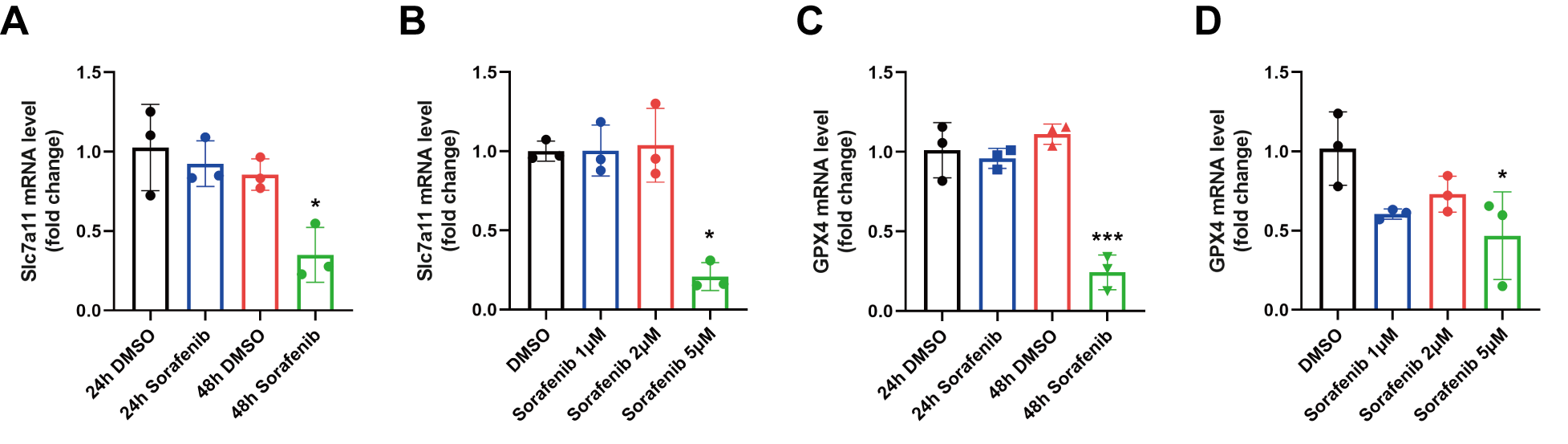

Supplement: Supplementary file 4 [file Image1.TIF]
